# Serum amyloid A contributes to radiation-induced lung injury by activating macrophages through FPR2/Rac1/NF-κB pathway

**DOI:** 10.7150/ijbs.100823

**Published:** 2024-09-16

**Authors:** Xinglong Liu, Yimeng Song, Songling Hu, Yang Bai, Jianghong Zhang, Guomei Tai, Chunlin Shao, Yan Pan

**Affiliations:** 1 Institute of Radiation Medicine, Shanghai Medical College, Fudan University, Shanghai 200032, China.; 2 Shanghai Institute of Infectious Disease and Biosecurity, Fudan University, Shanghai 200032, China.; 3 Department of Preventive Dentistry, Shanghai Key Laboratory of Craniomaxillofacial Development and Diseases, Shanghai Stomatological Hospital & School of Stomatology, Shanghai Medical College, Fudan University, Shanghai 200001, China.; 4 Department of Radiotherapy, Nantong Tumor Hospital and the Affiliated Tumor Hospital of Nantong University, Nantong 226631, Jiangsu Province, China.

**Keywords:** Thoracic irradiation, Lung injury, SAA, Macrophages, FPR2 and NF-κB

## Abstract

Patients who receive thoracic radiotherapy may suffer from radiation-induced lung injury, but the treatment options are limited as the underlying mechanisms are unclear. Using a mouse model of right thorax irradiation with fractionated doses of X-rays for three consecutive days (8 Gy/per day), this study found that the thoracic irradiation (Th-IR) induced tissue injury with aberrant infiltration of macrophages, and it significantly increased the secretion of TNF-α, IL-1β, IL-6, TGF-β1 and serum amyloid A (SAA) in mice. Interestingly, SAA could activate macrophages and then induce epithelial-mesenchymal transition (EMT) of lung epithelial cells and fibrosis progression in lung tissue. Mechanistically, SAA enhanced the transient binding of FPR2 to Rac1 protein and further activated NF-κB signaling pathway in macrophages. Inhibition of FPR2 significantly reduced pulmonary fibrosis induced by SAA administration in mice. In addition, cimetidine could reduce the level of SAA release after irradiation and attenuate the lung injury induced by SAA or Th-IR. In conclusion, our results demonstrated that SAA activated macrophages via FPR2/Rac1/NF-κB pathway and might contribute to the Th-IR induced lung injury, which may provide a new strategy to attenuate radiation-induced adverse effects during radiotherapy.

## Introduction

Radiotherapy constitutes part of the treatment of 40-50% of cancer patients, but radiation can also cause toxicity to normal tissues, such as inflammatory bowel disease, and cardiac and pulmonary toxicity 1. Radiation-induced lung injury (RILI) is one of the most serious complications during radiotherapy for thoracic malignancies, manifesting acutely as radiation pneumonitis (RP) and chronically as radiation-induced pulmonary fibrosis (RIPF) 2, 3. Research into effective interventions for this disease is urgently needed. However, the deterministic signaling factors involved in the RILI and its underlying mechanisms are still not fully understood.

Recently, it has been reported that RILI is closely related to macrophage activation and polarization, and that inflammatory factors are involved in the pathological progression 4. In particular, macrophages are the key effector cells for innate immunity amplification of RILI 5. Furthermore, epithelial cell damage and transformation in response to various stimuli is known to be the beginning of the progression of pulmonary fibrosis 6. However, the role of macrophage-epithelial cell interaction in RILI is still unclear.

For serum amyloid A (SAA), four genes on chromosome 11 have been identified to encode SAA 1, SAA2, SAA3, and SAA4 in the human genome. SAA1 and SAA2 proteins belong to a protein family of acute phase response, with SAA1 protein accounting for approximately 70% 7. SAA1/2 proteins are mainly synthesized in the liver but can also be produced in other tissues, such as stomach and intestine 8, which are the subtypes that this study mainly focuses on. The acute phase reactant SAA has been shown to mediate mutual interactions between the liver and joints, ultimately exacerbating chronic arthritis by enhancing macrophage activation 9. SAA may also have a protective effect. For example, it suppresses LPS-induced inflammation and lung injury in mice 10 and also plays an essential role in the immune-mediated inflammatory process, contributing to some chronic inflammatory diseases 11, 12. Moreover, SAA was increased in the serum of mice after irradiation and it was even proposed as a protein marker of radiation exposure 13, 14. Our previous study demonstrated that the SAA could be induced by thoracic irradiation (Th-IR) and caused bone marrow damage via stimulating ROS generation 15. However, the specific function of SAA in RILI, especially in chronic pulmonary fibrosis, remains unclear.

FPR2 is one of the primary receptors of SAA and widely expressed in the immune cells, including monocytes, neutrophils, and macrophages 16. Previous studies have shown that SAA stimulates inflammation 17, chemotaxis 18, invasion, and metastasis of tumor cells 19 by activating FPR2. As a pleiotropic receptor, FPR2 could mediate the signaling pathways of NF-κB 20, AMPK-mTOR 21, and TGF-β/Smad 22 after agonism. During NF-κB activation, as a small GTP-binding protein, Rac1 could generate a signaling complex with TLR2/4 23. However, the detailed interaction between SAA, FPR2 and Rac1 in the process of RILI is still obscure.

In this study, we found that irradiation of the right thorax of mice caused lung injury driven by SAA-associated cascade reaction responses. Mechanistically, SAA induced by thoracic irradiation activated macrophages via the FPR2/Rac1/NF-κB pathway, which ultimately induced fibrotic progression of lung epithelial cells. These findings suggest that the SAA/FPR2/NF-κB pathway may play a central role in RILI, accordingly, targeting these factors may have potential to alleviate the harmful effect of radiotherapy and improve the patient prognosis.

## Materials and Methods

### Animal and treatment

Male C57BL/6 J mice (6-8 weeks of age) were purchased from Shanghai SLAC Laboratory Animal Co. Ltd. All animals were hosted in the standard polypropylene cages with free access to food and water. Mice were randomly grouped into the sham-irradiation group (Sham-IR) and Th-IR group with 6-8 mice in each group. For irradiation, the right thorax of mice was locally exposed to 8 Gy X-rays per day with a dose rate of 0.883 Gy/min (X-RAD 320, PXI Inc., North Branford, CT, USA) for 3 consecutive days, and the rest parts of the mice were strictly covered with lead blocks. The mice of the Sham-IR group were covered with thick enough lead to shield X-rays. For SAA treatment, the mice were intraperitoneally injected with 0.35 mg/kg SAA (Catalog 300-13, PeproTech, Shanghai, China) on the first day, then 0.05 mg/kg SAA was intraperitoneally injected once a week for 4 weeks. In the SAA-induced injury model, mice were intraperitoneally injected with an FPR2-specific inhibitor, WRW4 (1.8 mg/kg) (S9818, Selleck, Houston, TX, USA), or administrated with cimetidine (CMTD) (100 mg/kg/day) 2 h before SAA treatment. In the radiation injury model, mice were administrated with CMTD (100 mg/kg/day) by gavage or intraperitoneally injected with an NF-κB specific inhibitor, BAY 11-7082 (10 mg/kg) (S2913, Selleck), from day 1 of Th-IR and continued until day 7 after Th-IR. All animal experiments were conducted according to the guidelines of the Declaration of Helsinki and approved by the Animal Welfare and Ethics Committee of Fudan University.

### Cell lines and treatment

Mouse capillary alveolar epithelial cell line MLE-12 and mouse mononuclear macrophage leukemia cell line RAW264.7 were purchased from ATCC and Shanghai Cell Bank (Shanghai, China), respectively. All cells were incubated in DMEM (Gibco, CA, USA) supplemented with 10% fetal bovine serum (Gibco, Thermo Fisher Scientific, Waltham, MA, USA) and 1% penicillin/streptomycin (Gibco) at 37 °C with 5% CO_2_.

To understand the effect of macrophages on lung epithelial cells, MLE-12 cells were cultured in the medium collected from RAW264.7 cells for 24 h, where the RAW264.7 cells were pretreated by 0 or 5 μg/mL SAA for 0, 2, 6, and 12 h. If necessary, RAW264.7 cells were treated with 10 μM BAY11-7082 (NF-κB inhibitor), 100 μM NSC33766 (Rac1 inhibitor), and 10 μM WRW4 (FPR2 inhibitor) for 12 h, 24 h and 2 h, respectively. All these inhibitors were purchased from Selleck (Huston, TX, USA).

### Cell proliferation detection

RAW264.7 macrophages were seeded in a 96-well plate at a density of 5 × 10^4^ cells per well. After cell growth was stable, cells were treated with various concentrations of SAA (0, 2, 5, 10 μg/mL) for 24 h. To determine the working concentrations of inhibitors, cells were treated with different concentrations of BAY11-7082 (0, 10, 20, 50, 100 μM) for 12 h, NSC33766 (0, 20, 50, 100, 200 μM) for 24 h, or WRW4 (0, 5, 10, 20, 50 μM) for 2 h. Then cell viability was detected with a CCK-8 Kit (C0038, Beyotime Biotechnology, Shanghai, China) following the manufacturer's protocol.

### Histological analysis

Paraffin-embedded lung tissue wax blocks were sectioned to 3-5 μm thick and performed H&E or Masson's trichrome staining. Then the tissue sections were blindly scored on a scale of 1-4 to evaluate the severity of alveolar inflammation and fibrosis. The detailed criteria are described in supplementary Table S1.

### Immunohistochemistry (IHC) assay and quantitative analysis

After deparaffinization and antigen repair, the sections were incubated with primary antibodies followed by secondary antibodies and then stained with DAB. For quantification of collagen deposition in Masson's trichrome staining and IHC staining of α-SMA (#19245, Cell Signaling Technology (CST), Boston, MA, USA), 8-10 fields of images were randomly selected to calculate the positive area by ImageJ software (version 18). For quantification of TGF-β1 (21898-1-AP, Proteintech, Wuhan, China) in lung tissues, the images were scored into 4 grades according to the intensity of the positive. The images were also rated as 4 grades according to the percentage of positive cells (Table S1). The final staining scores were obtained by multiplying the two scores.

### Immunofluorescence (IF) assay of tissue sections and quantitative analysis

Tissue sections were treated with the blocking buffer for 1 h and then incubated with diluted primary antibody overnight at 4℃. After washing three times with PBS, the sections were incubated with the fluorescein-conjugated secondary antibody for 1-2 h in the dark. Afterward, the sections were washed with PBS and stained with DAPI (Sigma-Aldrich, St. Louis, MO, USA). Finally, the tissues were sealed with nail polish and observed under a Leica DFC7000T microscope (Wetzlar, Germany). Primary antibodies used in the experiments included iNOS (#13120, CST), F4/80 (#70076, CST), CD206 (#24595, CST), Rac1-GTP (NB-26903, Biomol, Hamburg, Germany), and p-NF-κB p65 (#3033, CST). For quantification, 8-10 fields of images were randomly selected to calculate the positive area and fluorescence intensity by ImageJ software (version 18, National Institutes of Health, USA).

### Detection of inflammatory factors

Mice serum samples or cell culture medium were collected after conditioning and stored at -80°C until usage. The concentrations of TNF-α, SAA, TGF-β1, IL-6, and IL-1β were detected following the instructions of ELISA Kit (Lianke Biotech Co., Ltd, Zhejiang, China).

### Western blot assay

The protein extracts (15-40 µg) were separated by 10%-15% SDS-PAGE and transferred to a PVDF membrane. After 1 h blocking, the membrane was incubated with the primary antibody (1:1000) at 4℃ overnight and then incubated with the secondary antibodies (1:5000, Beyotime) at room temperature for 2 h. The protein bands were further treated with an ECL kit (Bio-Rad, Hercules, CA, USA) and quantified by Quantity One software (Bio-Rad). The primary antibodies included p-H2AX (Ser139) (#80312, CST), Vimentin (#46173, CST), α-Smooth Muscle Actin (#19245, CST), tubulin (Beyotime), N-Cadherin (#13116, CST), p-p38 (#4511, CST), p38 (#8690, CST), p-NFκB p65 (#3033, CST), NF-κB p65 (ab207297, abcam, Cambridge, UK), Rac1 (05-389, sigma-Aldrich), Rac1-GTP (NB-26903, Biomol), and FPR2 (P25090, Novus Biologicals, Littleton, CO, USA). For the detection of proteins with similar or the same molecular weight, after the first chemiluminescence, the original antibody was stripped and the membrane was incubated with other antibodies.

### ROS measurement

The intracellular ROS level was measured with a ROS detection kit (S0033S, Beyotime). RAW264.7 macrophages were treated with 10 μM 2,7-dichlorohydro-fluorescin diacetate at 37 °C for 30 min and then washed with PBS. The fluorescence intensity with detected with a flow cytometry (CytoFLEX, Beckman Coulter, CA, USA).

### Cell cycle analysis

RAW264.7 macrophages were collected and fixed with 70% ethanol. After 24 h of storage at -20 ℃, the cells were washed with PBS and stained with a cell cycle regent (BD Biosciences). The cell cycle distribution was detected with a flow cytometry and analyzed with FlowJo software (version 10).

### Cell immunofluorescence (IF) assay

Cells were washed twice with PBS and fixed in 4% paraformaldehyde for 15 min. After permeabilization with Triton for 15 min, the cells were incubated in the blocking solution for 30 min and then labeled with primary antibody overnight at 4°C. Nuclei were stained with DAPI (GDP1024, Servicebio, Wuhan, China) for 10 min after the incubation of secondary antibody. The primary antibodies included F-actin (ab130935, abcam), Arg-1 (#89872, CST), iNOS (#13120, CST), Vimentin (#46173, CST), FPR2 (P25090, Novus Biologicals), Rac1-GTP (NB-26903, Biomol). Secondary antibodies were goat anti-mouse IgG H&L (Alexa Fluor® 488) (ab150113, abcam) and goat anti-mouse IgG H&L (Alexa Fluor® 555) (ab150114, abcam). Finally, the cell fluorescence image was viewed under an ImageXpress Micro 4 screening system (Molecular Devices, Sunnyvale, CA, USA) and analyzed using ImageJ software.

### Co-immunoprecipitation (Co-IP) assay

RAW264.7 macrophages were harvested at the indicated time points after treatments, and incubated with moderate RIPA lysis buffer (P0013C, Beyotime) containing 1% PMSF and 1% cocktail for 30 min on ice. Lysates were collected by centrifugation (12000 g, 10 min) and the protein concentration was determined with a BCA kit (P0012S, Beyotime). Then 500 μg protein was used for Co-immunoprecipitation assay. Anti-Rac1 antibody (05-389, Sigma) was used as the precipitating antibody, and Anti-FPR2 (P25090, Novus Biologicals) were used for Western blot analysis.

### RNA sequencing

RAW264.7 macrophages on 80% confluence were treated with 0 or 5 μg/mL SAA for 6 h. Total RNA was extracted by using a cool Trizol reagent (Invitrogen, Carlsbad, CA, USA) and transferred to an RNA enzyme-free EP tube. The RNA sequencing was performed by Huada Gene Technology Co., Ltd. Reference species was Mus_musculus from NCBI and the reference genome version for comparison was GCF_000001635.26_GRCm38.p6. Heatmaps and volcano plots were drawn using the ggplot2 package, and enrichment analysis was performed with clusterProfiler package in R software (version 4.3.1).

### Tandem Mass Tagging (TMT) proteomics analysis

The serum samples were collected from the mice in Sham-IR, Th-IR, and Th-IR+CMTD groups on the 7th day after irradiation. High abundant plasma proteins were removed by using the high-abundance affinity column Mouse 3 to obtain the effective component solutions. The protein purity was determined by SDS-PAGE electrophoresis assay, and then the proteins were performed with pre-experiment to ensure protein quality. Finally, LC-MS/MS analysis for the serum proteins was carried out by Genechem Co. Ltd (Shanghai, China). The experimental procedures were described in a previous study 15.

### Statistical analysis

All experiments were repeated at least three independent experiments and results are presented as mean ± standard error (S.E.). Comparisons were assessed by Student's t-test between two groups and by one-way analysis of variance (ANOVA) among two more groups. *p*< 0.05 was considered statistically significant.

## Results

### Th-IR induced pneumonia and fibrosis in mouse lung tissue

**Figure 1:**
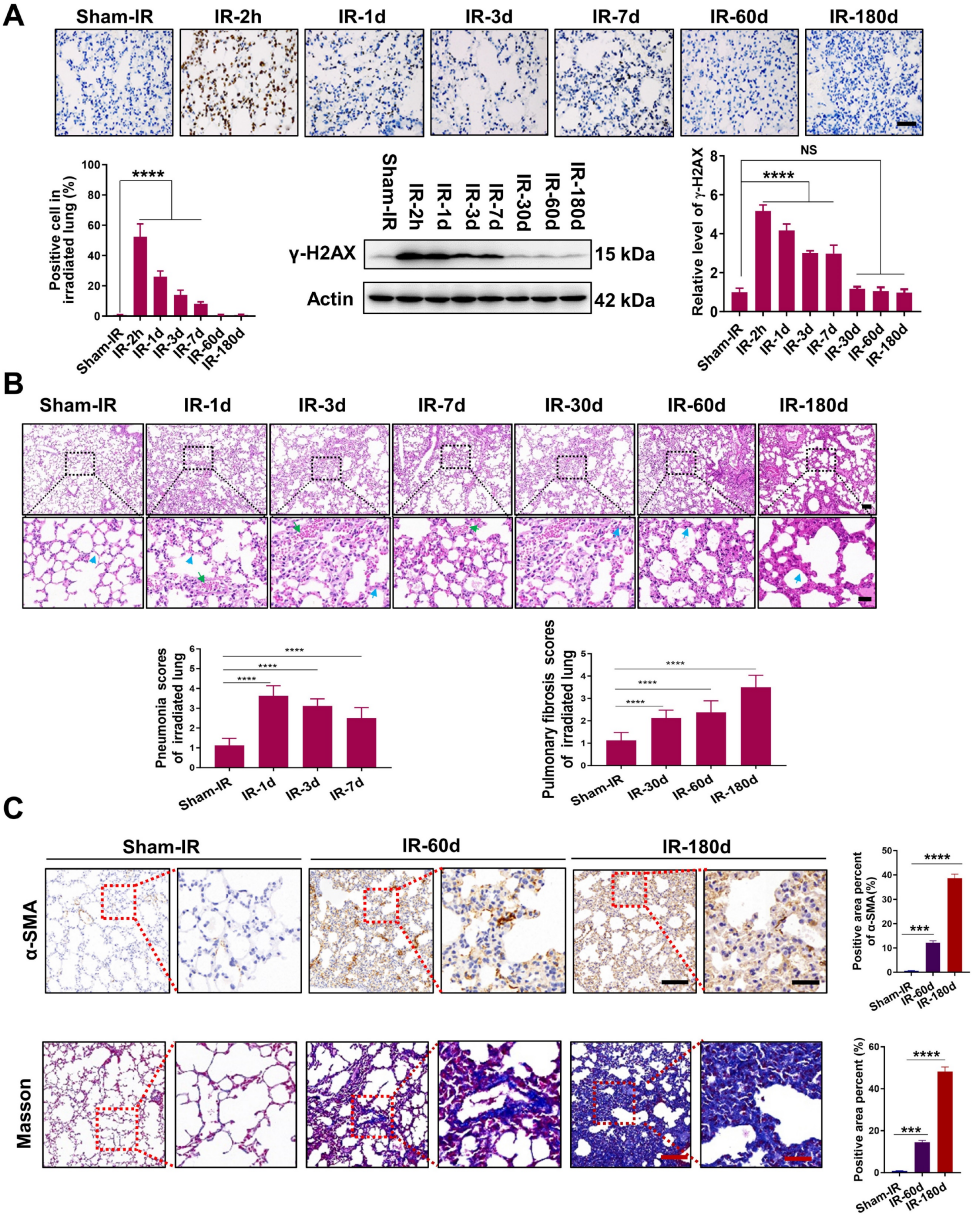
**Th-IR induced tissue damage in the irradiated lung tissues of mice**. (A) IHC staining and western blot assay for γ-H2AX proteins in lung. (B) H&E staining images and quantitative analysis of pneumonia and fibrosis of lung. Blue arrows indicate the thickened alveolar wall and green arrows indicate hemorrhage exudate. (C) IHC staining for α-SMA and Masson's trichrome staining of lung at 60 and 180 days after Th-IR. Scale bar=150 μm, magnifying scale bar=50 μm. n=6-8 for each group, ** *p* < 0.01, *** *p* < 0.001, **** *p* < 0.0001 compared with Sham-IR group. NS, no significant difference.

To evaluate the lung tissue injury induced by Th-IR, we established an animal model in which the right lung of the mice was locally irradiated with fractionated doses of X-rays for three consecutive days (8 Gy/per day). The irradiated lungs of the mice were collected from 2 h to 180 days after Th-IR for further analysis (Fig. S1A). It was found that the formation of γ-H2AX was significantly increased in the irradiated lung of mice with a maximum value of 58.9 fold of the control at 2 h after Th-IR (Fig. 1A). H&E staining showed that the lung structure was altered within 7 days after Th-IR (most severe on day 1), manifested by severe interstitial congestion and edema (green arrows) and thickened alveolar walls (blue arrows) accompanied by inflammatory cell infiltration (Fig. 1B). Fibrosis-related symptoms were detected in irradiated lung tissues after 60-180 days of Th-IR, characterized by a significant increase in fibroblasts and thickness of alveolar walls (blue arrows) (Fig. 1B). IHC staining for α-SMA and Masson's trichrome staining also showed that the collagen deposition in the irradiated lung tissues was increased with time after Th-IR (Fig. 1C). The positive area of α-SMA increased from 0.59% to 38.58% and the positive area of collagen increased from 0.78% to 48.19% at 180 days after Th-IR.

### Th-IR promoted macrophage infiltration and cytokine expression

**Figure 2:**
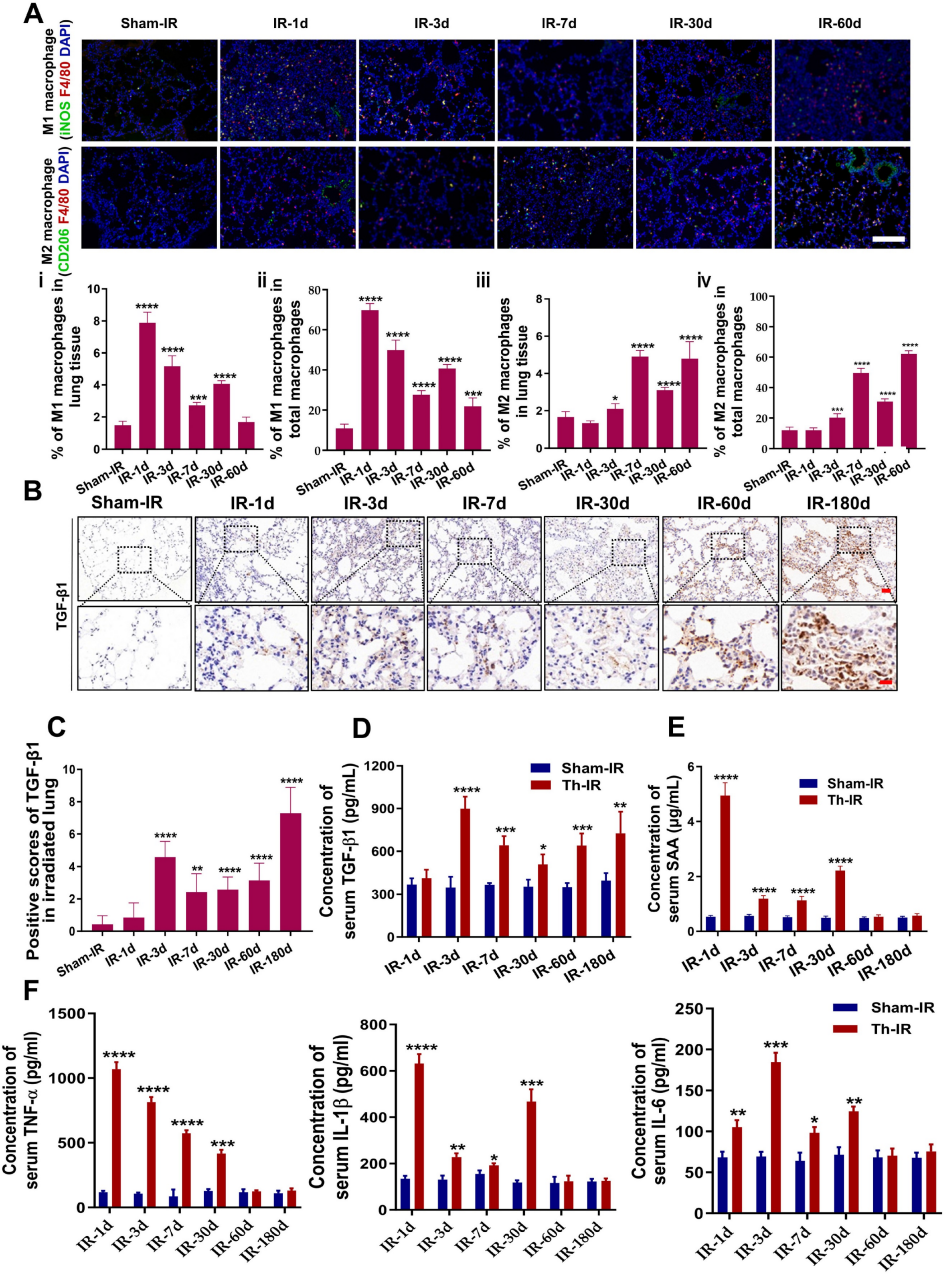
** Th-IR affected the macrophage distribution and phenotypes in lung and cytokine expression**. (A) IF staining images of macrophages in lung. (ⅰ, ⅲ) Proportion of M1/M2 macrophages over the lung tissues; (ⅱ, ⅳ) Proportion of M1/M2 macrophages over the total number of macrophages in lung. Scale bar=100 μm. (B-C) IHC staining (B) and positive scores for TGF-β1 expression (C) in lung. Scale bar=150 μm, magnifying scale bar=50 μm. (D-F) The concentrations of TGF-β1 (D) SAA (E), TNF-α, IL-1β, and IL-6 (F) in mouse serum after Th-IR. n=6-8 for each group, * *p* < 0.05, ** *p* < 0.01, *** *p* < 0.001, **** *p* < 0.0001 compared with Sham-IR group.

Pulmonary macrophages are one of the major cell clusters in the lung tissue and also are crucial mediators of the immune response to lung injury. IF staining for F4/80 (macrophages), iNOS (M1), and CD206 (M2) showed that the infiltration of M1 macrophages increased rapidly at day 1 after Th-IR and then decreased over time in the irradiated mouse lung, while the infiltration of M2 macrophages could be detected over a long term (Fig. 2A).

TGF-β1 is an important pro-fibrotic cytokine that plays a pivotal role in the development of fibrosis. Th-IR also increased the expression of TGF-β1 in the irradiated lung tissues, which peaked at 180 days (Fig. 2B-C). The level of serum TGF-β1 was elevated in the meantime with a similar time trend, peaking at day 3 after Th-IR (Fig. 2D). Besides, the contents of pro-inflammatory factors including SAA, TNF-α, IL-6, and IL-1β were also significantly increased in the serum of mice shortly after Th-IR and maintained at high levels up to 30 days (Fig. 2E-F). Accordingly, Th-IR promoted macrophage infiltration into the irradiated lung tissue and induced inflammation-related cytokine expressions in mice.

### SAA facilitated EMT process of epithelial MLE-12 cells by aviating macrophages

**Figure 3:**
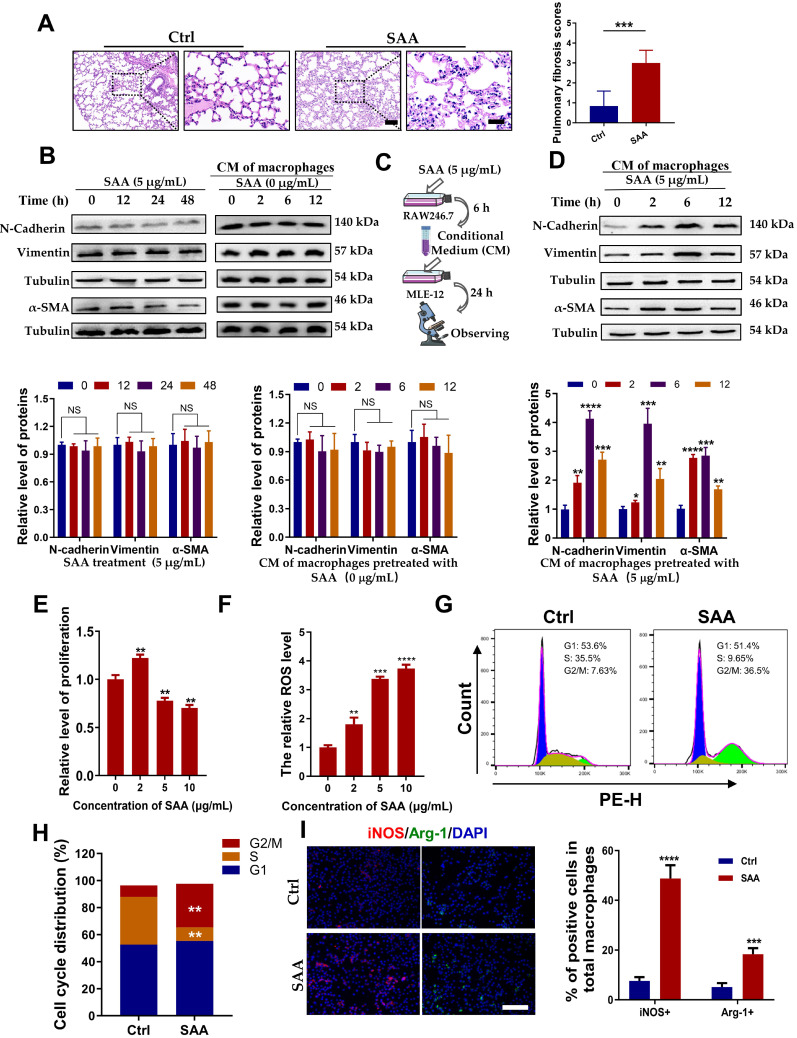
**SAA promoted EMT progression in lung epithelial cells by activating macrophages**. (A) H&E staining of lung tissues at 10 weeks after the first SAA injection. Scale bar=150 μm, magnifying scale bar=50 μm. (B-D) Expression of indicated proteins in MLE-12 cells that were treated with 5 μg/mL SAA for 0, 12, 24, and 48 h (B) or incubated with the conditioned medium of macrophages for 24 h, where the macrophages were pretreated by 0 μg/mL (C) or 5 μg/mL SAA for 0, 2, 6, and 12 h (D). (E-F) Relative proliferation level (E) and ROS level (F) of RAW264.7 macrophages treated with SAA. (G-H) Representative images (G) and cell cycle distribution (H) of RAW264.7 macrophages treated with or without SAA. (I) IF staining for iNOS (red), Arg-1 (green), and DAPI (blue) in RAW264.7 cells treated with/without SAA for 6 h. Scale bar=100 μm. * *p* < 0.05, ** *p* < 0.01, *** *p* < 0.001, **** *p* < 0.0001 between indicated groups.

Our previous study demonstrated that Th-IR increased the level of serum SAA which mediated the abscopal damage to bone marrow cells 15, and hence we want to know whether SAA is also a key factor contributing to the lung injury after Th-IR. According to the determined serum SAA level (Fig. 2E), the mice were intraperitoneally injected with 0.35 mg/kg SAA on the first day and then with 0.05 mg/kg SAA once a week for 4 weeks (Fig. S1B). H&E staining showed that the thickness of the alveolar wall was increased at week 10 after the first injection of SAA (Fig. 3A), demonstrating that exogenous administration of SAA could induce lung injury.

To clarify how SAA promotes lung tissue injury, we treated mouse lung epithelial MLE-12 cells with SAA directly or with the conditioned medium (CM) generated from RAW264.7 macrophages. Surprisingly, neither SAA nor CM treatment altered the expressions of the hallmarks of epithelial-mesenchymal transition (EMT) (N-cadherin and vimentin) and fibrosis (α-SMA) in MLE-12 cells (Fig. 3B). However, when RAW264.7 macrophages were pre-treated with SAA, their CM remarkably increased the expressions of EMT-related proteins and α-SMA in MLE-12 cells (Fig. 3C-D), implying that the promotion of macrophages to the EMT process of epithelial cells is SAA dependent. Indeed, SAA at a certain concentration (≥5 μg/mL) inhibited the proliferation and increased the intracellular ROS level of RAW264.7 macrophages (Fig. 3E-F). Consistent with the cell proliferation assay, 5 μg/mL SAA induced cell cycle arrest in RAW264.7 macrophages (Fig. 3G-H). We then investigated whether SAA could affect macrophage polarization *in vitro*. It was found that treatment of RAW264.7 cells with SAA for 6 h increased the expression of iNOS (M1 marker) by 51.11% and Arg-1 (M2 marker) by 18.12% (Fig. 3I), demonstrating that SAA did mainly polarize macrophages to M1. Collectively, SAA might promote the EMT process of epithelial MLE-12 cells by aviating macrophages.

### SAA activated FPR2/Rac1/NF-κB pathway in macrophages

**Figure 4:**
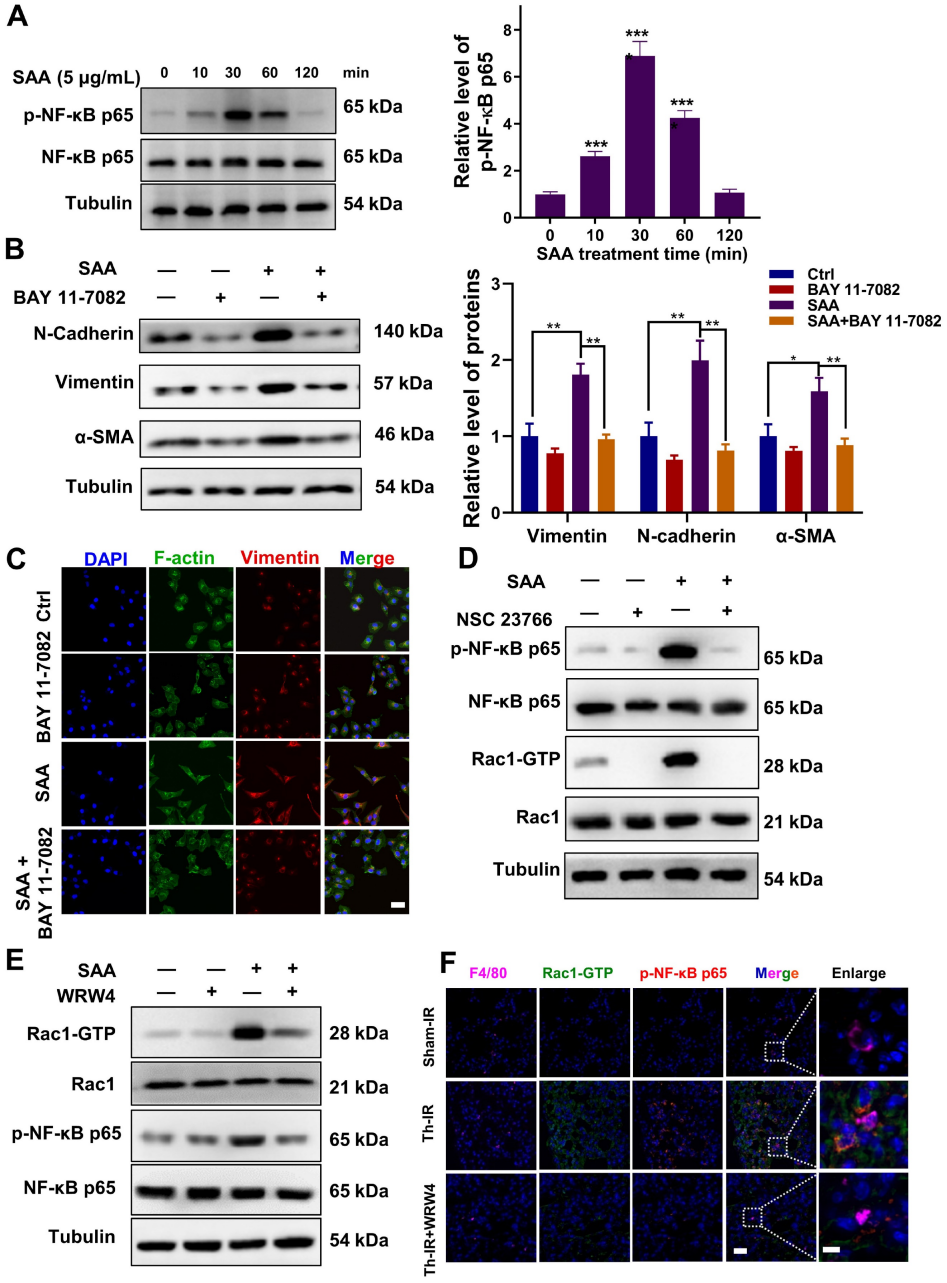
** SAA activated FPR2/Rac1/NF-κB pathway in RAW264.7 macrophages**. (A) Expression of NF-κB/p-NF-κB protein in macrophages measured by western blot assay. (B-C) Expression of indicated proteins (B) and IF staining of F-actin (green) and Vimentin (red) (C) in MLE-12 cells incubated for 24 h with the conditioned medium of macrophages pretreated with or without NF-κB inhibitor of BAY 11-7082 (2 μM) for 12 h. Bar=50 μm. (D-E) Expressions of indicated proteins in macrophages pretreated with NSC23766 (Rac1 inhibitor) (D) and WRW4 (FPR2 inhibitor) (E) for 24 h and 2 h, respectively, before SAA treatment. (F) IF staining for F4/80 (purple), Rac1-GTP (green), and p-NF-κB p65 (red) in mouse lungs on day 1 after Th-IR and WRW4 treatment. The mice were injected intraperitoneally with FPR2 inhibitor WRW4 for 2 h before irradiation, and lung tissues were collected at 1 day after Th-IR (n=6-8 for each group). Scale bar=30 μm, magnifying scale bar=10 μm. * *p* < 0.05, ** *p* < 0.01, *** *p* < 0.001 between indicated groups.

Furthermore, we explored how SAA activated macrophages to promote the EMT process. SAA has been reported to stimulate NF-кB activation in human hepatocellular liver carcinoma HepG2 cells 24. We also found that SAA treatment increased the expression of p-NF-κB in RAW264.7 macrophages (Fig. 4A). Pre-treatment of RAW264.7 macrophages with BAY-117082, an inhibitor of NF-κB, alleviated CM-induced EMT process and morphological changes in MLE-12 cells (Fig. 4B-C). The working concentrations of indicated inhibitors, including BAY-117082 (10 μM), NSC23766 (100 μM), and WRW4 (10 μM), did not affect cell proliferation of macrophages (Fig. S2A-C). BAY-117082 administration also alleviated the fibrosis phenotype in the irradiated lung at week 8 after Th-IR (Fig. S3A-B). This suggested that SAA might mediate the EMT process of lung epithelial cells by activating the NF-κB pathway in macrophages.

To further understand how SAA activated RAW264.7 macrophages through NF-κB pathway, RNA sequencing was performed in RAW264.7 cells with or without SAA treatment. A total of 181 genes (110 up-regulated and 71 down-regulated) (fold change > 2) were defined as differentially expressed genes (DEGs) between the SAA treatment and control groups (Fig. S4A, Table S2). The DEGs were mainly enriched in immune function-related pathways such as cytokine-cytokine receptor interaction and TNF signaling pathway (Fig. S4B).

Remarkably, the genes of the small GTPase family (Cdc42ep2, etc.) were altered after SAA treatment, implying that small GTP-binding proteins may regulate cellular functions in macrophage activation (Fig. S4C). The GTP-binding protein Rac1 was reported to be capable of activating multiple signaling pathways, including ROS generation and NF-κB, MAPK, and STAT3 25, 26, thus we examined whether SAA could modulate the NF-κB signaling pathway through Rac1 in macrophages. Indeed, SAA activated Rac1 by increasing the expression of Rac1-GTP, the active binding form of Rac1 protein, in macrophage cells, whereas SAA-activated p-NF-κB was suppressed by NSC23766, an inhibitor of Rac1-GTP (Fig. 4D, S5A).

Similarly, when FPR2 (one of the SAA receptors) was inhibited by WRW4, the SAA-induced Rac1-GTP expression was significantly reversed, and the activation of p-NF-κB by SAA was also remarkably inhibited in macrophages (Fig. 4E, S5B). Accordingly, FPR2 might be a significant regulator of fibrotic progression. To verify this assumption *in vivo*, mice were injected intraperitoneally with the FPR2 inhibitor WRW4 before Th-IR. It was found that the Rac1 and NF-κB pathways were significantly activated in macrophages (F4/80+) of mice lungs after Th-IR, which was reduced by WRW4 (Fig. 4F). Taken together, SAA activated the Rac1/NF-κB signaling in macrophages via its receptor FPR2, further promoting the EMT process of pulmonary epithelial cells.

### SAA promoted the binding of FPR2 with Rac1 in macrophages

**Figure 5:**
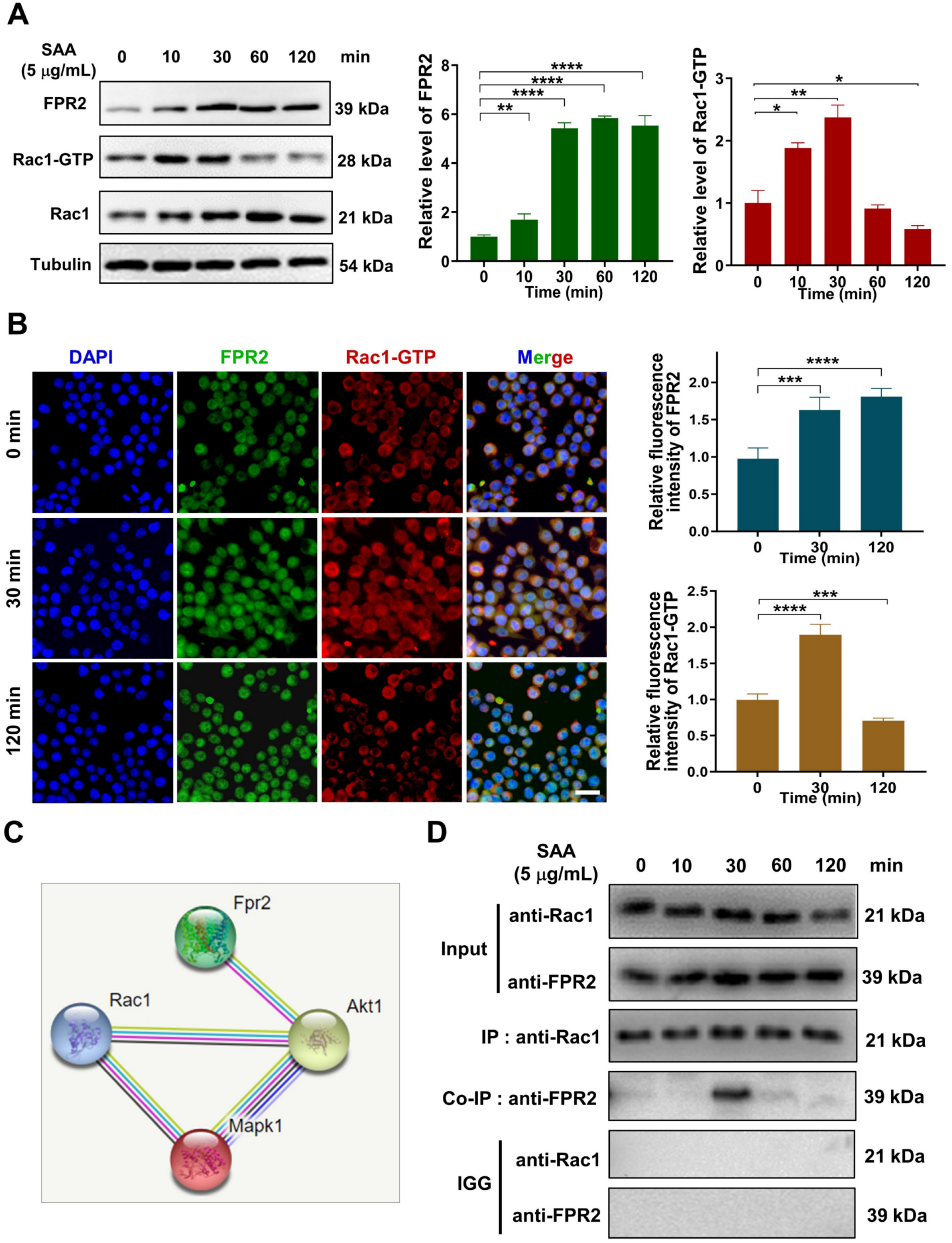
** SAA promoted protein binding of FRP2 and Rac1 in RAW264.7 macrophages**. (A) Protein expression of FRP2 and Rac1-GTP in macrophages after the cells treated by SAA at different time points. (B) IF staining for FRP2 and Rac1-GTP protein in RAW264.7 macrophages. Scale bar=50 μm. (C) Online STRING analysis of protein-protein interaction among FPR2, Rac1, and Akt. (D) Co-IP assay of FRP2 and Rac1 protein in macrophages treated by SAA. * *p* < 0.05, ** *p* < 0.01, *** *p* < 0.001, **** *p* < 0.0001 between indicated groups.

How does FPR2 transduce the signals that stimulate the fibrotic process? We found that SAA treatment up-regulated the expressions of FPR2 and Rac1 in macrophages, and a co-expression trend of these two proteins was observed within 30 min after SAA administration (Fig. 5A-B). Thus, we speculated that there might be a cross-interaction between FPR2 and Rac1, although no direct relationship could be predicted by the STRING online tool (version 11.0) (Fig. 5C). Co-IP assay demonstrated the binding of FPR2 to Rac1 in macrophages at 30 min after SAA treatment, but this binding vanished at 60 min after SAA treatment, showing a rapid binding dissociation pattern (Fig. 5D). Collectively, SAA promoted the binding of FPR2 and Rac1, thereby activated macrophages to promote the pro-fibrotic process.

### CMTD alleviated lung injury induced by Th-IR

**Figure 6:**
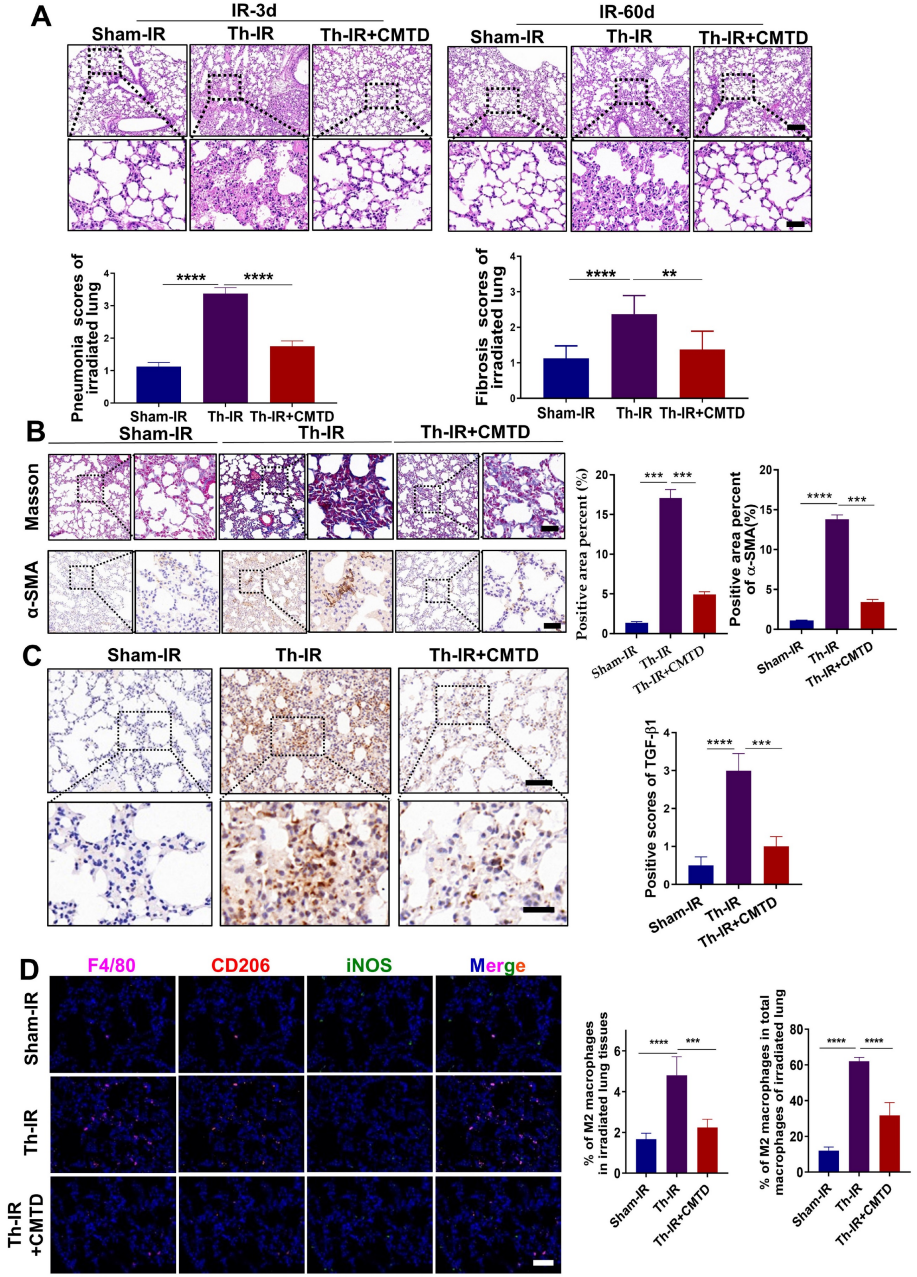
**CMTD alleviated lung injury induced by irradiation**. The mice were given CMTD (100 mg/ kg/day) by oral gavage from the first day of Th-IR and continued to 7 days after Th-IR. (A) H&E staining of lung and quantitative analysis of injury of lung at day 3 and day 60. (B) Masson trichrome staining and IHC staining for α-SMA of lung at day 60 after Th-IR. (C) IHC staining for TGF-β1 of lung at day 60 after Th-IR. Scale bar=150 μm, magnifying scale bar=50 μm. (D) IF staining for F4/80 (purple), iNOS (green), CD206 (red), and DAPI (blue) in mouse lung at day 60 after Th-IR. Scale bar=100 μm. n=6-8 for each group, ** *p* < 0.01, *** *p* < 0.001, **** *p* < 0.0001 between indicated groups. NS, no significant difference.

We have previously reported that CMTD can ameliorate Th-IR-induced abscopal damage in the testis 27. We then wondered whether CMTD has a protective role against Th-IR induced lung injury, so the mice were treated with CMTD by oral gavage. HE staining of the lung tissues of mice showed that CMTD alleviated the massive hemorrhage and inflammatory cell infiltration caused by Th-IR within 3 days, and mitigated the thickened alveolar wall and widened alveolar septum after Th-IR in a long term (60 days) (Fig. 6A). Masson's trichrome staining and IHC staining for TGF-β1 and α-SMA also proved that CMTD significantly reduced the fibrosis and collagen expressions in the irradiated lungs (Fig. 6B-C). Moreover, CMTD also effectively reduced the distribution of M2 macrophages in the irradiated lung (Fig. 6D), indicating that CMTD may have an anti-fibrosis effect during irradiation. In addition, TMT analysis for serum proteins revealed that CMTD partially reduced the protein changes induced by Th-IR at day 7 after irradiation, especially for the SAA protein (Fig. S6, Table S3), suggesting that the protective role of CMTD against Th-IR-induced lung injury may be achieved by inhibiting the secretion of SAA protein.

### CMTD relieved SAA-induced pulmonary fibrosis in mice

**Figure 7:**
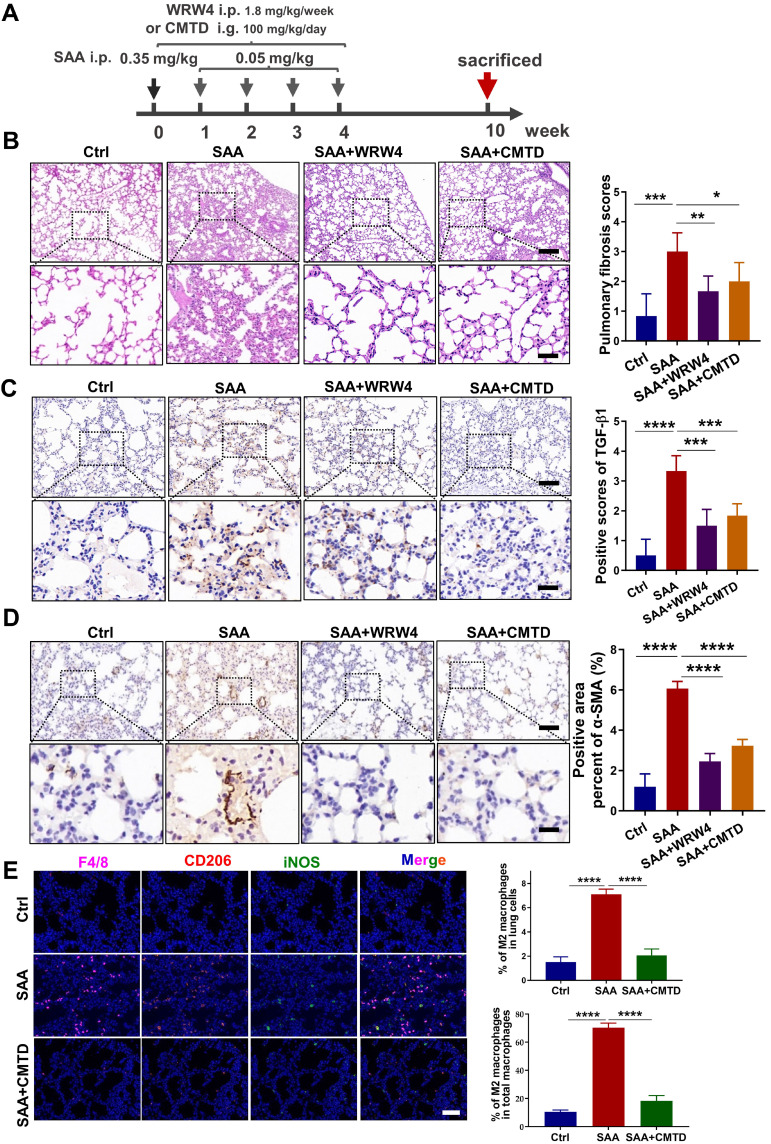
**Inhibitor of FPR2 or CMTD alleviated lung injury induced by SAA**. (A) Schematic flow of WRW4 or CMTD treatment in SAA-indued lung injury mouse model. The mice were given an FPR2 inhibitor (WRW4, 1.8 mg/kg) via intraperitoneal injection or CMTD (100 mg/kg/day) by gavage at 2 h before SAA treatment. (B-D) H&E staining (B) and IHC staining for TGF-β1(C) and α-SMA (D) of lung tissue at 10 weeks after the first SAA injection. Scale bar=150 μm, magnifying scale bar=50 μm. (E) IF staining for F4/80 (purple), iNOS (green), CD206 (red), and DAPI (blue) in mouse lungs treated with/without SAA at 10 weeks after the first SAA injection. Scale bar=100 μm. n=6-8 for each group, * *p* < 0.05, ** *p* < 0.01, *** *p* < 0.001, **** *p* < 0.0001 between indicated groups.

To further confirm the relationship between the protective effect of CMTD and the profibrotic effect of SAA *in vivo*, the mice were gavaged with CMTD prior to injection of SAA proteins (Fig. 7A). It was found that SAA-increased thickness of alveolar walls was alleviated by the pre-injection of CMTD (Fig. 7B), and SAA-increased expressions of α-SMA and TGF-β1 in lung tissues were also attenuated by CMTD administration (Fig. 7C-D). Interestingly, when WRW4 was intraperitoneally injected 2 h before SAA treatment in mice, it had a similar protective effect as CMTD during SAA treatment (Fig. 7A-D). In addition, SAA injection increased M2 macrophage infiltration in the lung, which was diminished by CMTD (Fig. 7E). Taken together, SAA could induce a pulmonary fibrosis phenotype via FPR2, and CMTD had a significant protective effect against radiation- or SAA-induced lung injury.

## Discussion

**Figure 8:**
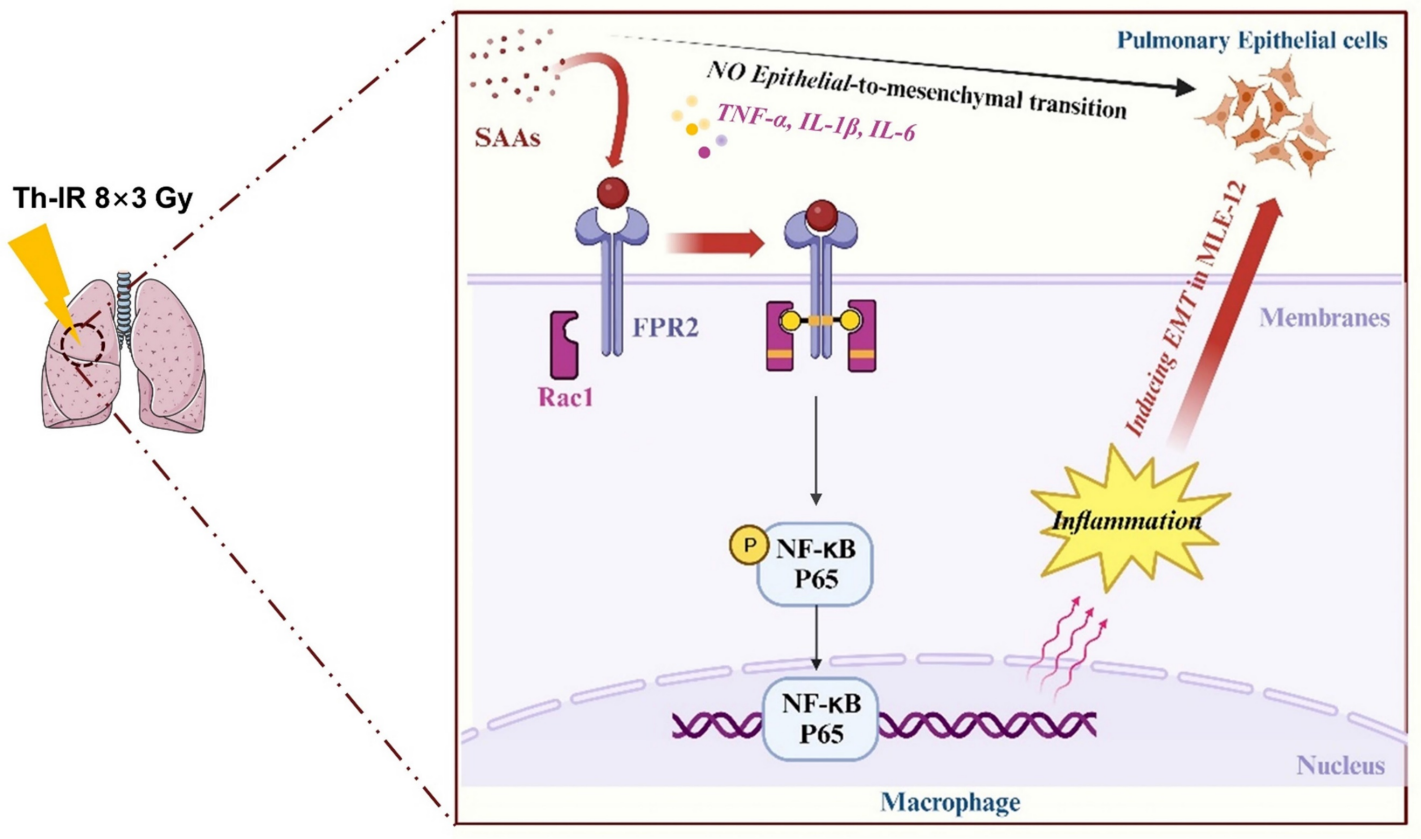
** Schematic mechanism of thoracic irradiation inducing lung injury via the SAA/FRP2/Rac1/NF-κB pathway**. Fractionated irradiation of the right thorax triggers the cytokine production and up-regulates serum SAA level, which induces EMT and fibrosis process of lung epithelial cells via activation of the FPR2/Rac1/NF-κB pathway in macrophages.

Accumulating studies are focused on the development of strategies to minimize the side effects of cancer radiotherapy on normal tissues. Lung injury is inevitable during chest radiotherapy. We found that Th-IR triggered SAA generation and promoted fibrosis progression via the FPR2/Rac1/NF-κB pathway of macrophages in mouse lungs (Fig. 8), providing a potential molecular target for mitigating the side effects of thoracic radiotherapy.

Macrophages are the innate immune cells that can maintain lung homeostasis and function throughout the pathological process of RILI. In the early stage of RILI, macrophages could be recruited to the injured lung tissues and undergo polarization into M1 phenotypes, inducing an inflammatory storm by secreting more pro-inflammatory cytokines (TNF-α, IL-1, etc.) and promoting the development of RP 28, 29. M1 macrophages could cause lung tissue damage by increasing the expression of iNOS or the production of ROS that directly induced DNA damage 30, 31. Whereas, M2 macrophages were increased in the late stage of pulmonary fibrosis 32. Similarly, we found that the irradiated lung harbored predominantly M1 macrophages within 3 days and increased to two-fold of control at 30 days after Th-IR. M2 macrophages were largely infiltrated into the irradiated lung at a delayed time and peaked at the mid- and late-fibrosis stage. This reflects a potential relationship between macrophage polarization and RILI.

Furthermore, we found that SAA was a possible culprit in RILI. As a major acute-phase protein, SAA is mainly produced by hepatocytes during acute-phase response and then released into blood with a subtype of SAA1/2 33. The exact role of serum SAA in the progression of diseases remains controversial. On the one hand, SAA has been implicated in triggering hepatic steatosis and intrahepatic inflammatory response by forming an SAA/TLR4/NF-κB/SAA feed-forward regulatory circuit 34. On the other hand, SAA deficiency has been reported to exacerbate sepsis-induced mortality and lung injury in mice by impairing neutrophil transmigration into the injured lung 35. After irradiation, the serum SAA concentration was rapidly increased within a few hours and then gradually decreased to baseline levels within three days in mice 36. However, in this study, we found that the serum SAA level peaked at 1 day after Th-IR and remained at a higher level within 30 days after Th-IR, indicating that the Th-IR induced a relatively longer period of SAA activation in mice.

SAA has been implicated in the development of amyloidosis, inflammatory bowel disease, lung disease, and even cancers 37, 38. For instance, SAA promotes the migration of ovarian cancer OVCAR-3 cells by regulating MMPs and EMT 39. Interestingly, we found that SAA alone did not affect the EMT progression of MLE-12 cells directly, but it could activate macrophages to release inflammatory factors and then promote the EMT process of MLE-12 cells. This suggests that the function of SAA maybe different between normal and tumor cells. SAA can dramatically induce the secretion of cytokines including IL-1β, MCP-1, IL-6, and TNF in monocyte-derived macrophages, leading to chronic inflammation 40, 41. Our *in vivo* study showed that SAA injection increased fibrosis formation in the lung of mice. Consistently, a previous statistical analysis demonstrated that SAA might be a promising marker of disease severity in patients with idiopathic pulmonary fibrosis 42. These results suggest that SAA may be a potential therapeutic target for pulmonary fibrosis.

Moreover, we found that Th-IR-induced SAA protein expression in mouse serum could be partially reduced by CMTD, a histamine type II receptor blocker, and that pulmonary inflammation and fibrosis induced by Th-IR and SAA were also mitigated by CMTD administration. Similarly, CMTD has been reported as a promising radioprotective agent through modulating Bax/Bcl2 ratio in rat model 43. Together, it is suggested that CMTD administration may be clinically helpful in reducing the side effects during radiotherapy of lung cancer.

In addition, the role of SAA on cell responses depends on the activation of its receptors, including TLR2, TLR4, FPR2, SR-B1, RAGE, CLA-1, and CD36 8, 44. Sandri *et al.* found that SAA aviated macrophage via receptor TLR4 in mice 45. Li *et al.* also found SAA induced HMGB1 release via receptors TLR4 and RAGE in macrophages 46. It seems that various receptors contribute to macrophage aviation. Among these receptors, FPR2 is thought to have pleiotropic effects due to its unique protein structure 47. Upon binding to its ligand, FPR2 immediately activates GTPases of Ras superfamily and other signaling pathways such as TGF-β/Smad, AKT, and NF-κB 48-50. We found that SAA mediated RILI through its receptor FPR2, and SAA significantly affected the Rac1/NF-κB pathway via FPR2 in macrophages. And FPR2 inhibitor reduced Th-IR induced activation of the Rac1/NF-κB pathway in macrophages and further alleviated SAA-induced lung fibrosis. It was also reported that lipoxin A4-FPR2 signaling could attenuate RIPF through the crosstalk with TGF-β/Smad signaling 51. Especially, we found that SAA promoted a transient association between FRP2 and Rac1, further deepening the understanding of the role of FPR2 in macrophage activation during RILI. Overall, this study has broadened the understanding of the role of the acute protein SAA in tissue injury, and targeting SAA shows promise for reducing the side effects of chest radiotherapy. Our results further validated the potential of CMTD as a radioprotective drug, providing more evidence for clinical implication.

## Conclusions

This study provides the first evidence that SAA participates in Th-IR induced lung injury through signal transduction between epithelial cells and macrophages in lung tissue, and SAA/FPR2/Rac1/NF-κB signaling pathway may represent novel therapeutic targets for alleviating RILI in clinical practice.

## Supplementary Material

Supplementary figures.

Supplementary tables.

## Abbreviations

RILI: radiation-induced lung injury
RP: radiation pneumonitis
RIPF: radiation-induced pulmonary fibrosis
Th-IR: thoracic irradiation
SAA: serum amyloid A
CMTD: Cimetidine
EMT: epithelial-mesenchymal transition
DEGs: differentially expressed genes
Sham-IR: sham-irradiation
