# Supplementary material for: Serum amyloid A contributes to radiation-induced lung injury by activating macrophages through FPR2/Rac1/NF-κB pathway
Source: Int J Biol Sci. 2024 Sep 16;20(12):4941–56. doi: 10.7150/ijbs.100823 (PMC11414394; doi:10.7150/ijbs.100823)
Supplement: Supplementary file 1 — Supplementary figures. [file ijbsv20p4941s1.pdf]

Serum amyloid A contributes to radiation-induced lung injury by activating macrophages through FPR2/Rac1/NF-κB pathway

Xinglong Liu[1,2*], Yimeng Song[1,*], Songling Hu[3,*], Yang Bai[1], Jianghong Zhang[1], Guomei Tai[4,#], Chunlin Shao[1,#], Yan Pan[1,#]

[1]Institute of Radiation Medicine, Shanghai Medical College, Fudan University, Shanghai 200032, China

[2]Shanghai Institute of Infectious Disease and Biosecurity, Fudan University, Shanghai 200032, China.

[3]Department of Preventive Dentistry, Shanghai Key Laboratory of Craniomaxillofacial Development and Diseases, Shanghai Stomatological Hospital & School of Stomatology, Shanghai Medical College, Fudan University, Shanghai 200001, China.

[4]Department of Radiotherapy, Nantong Tumor Hospital and the Affiliated Tumor Hospital of Nantong University, Nantong 226631, Jiangsu Province, China.

* These authors contributed equally to this work.

# Corresponding authors:

Dr. Yan Pan, E-mail: swallowpan@fudan.edu.cn; Prof. Chunlin Shao, E-mail: clshao@shmu.edu.cn; Dr. Guomei Tai, E-mail: guomeitai@163.com.

Supplementary figures

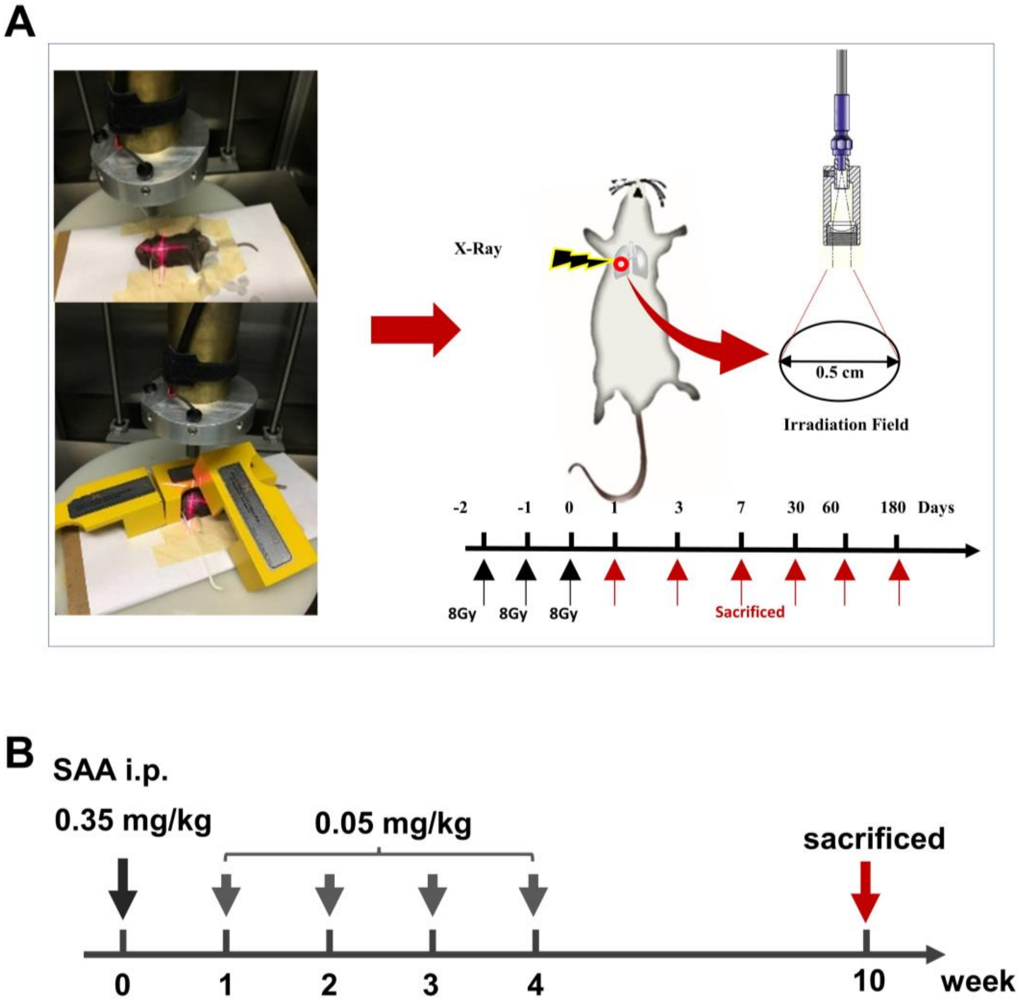

Figure S1. A Thoracic irradiation model in mice. The right lung of the mice was irradiated by X-rays with 8 Gy each day for three days through a circular collimator. **B** Schematic flow of SAA-induced lung injury mouse model. The mice were intraperitoneally injected with 0.35 mg/kg SAA on the first day and then with 0.05 mg/kg SAA repeated once a week for 4 weeks, lung tissues were collected at 10 weeks after the first SAA injection.

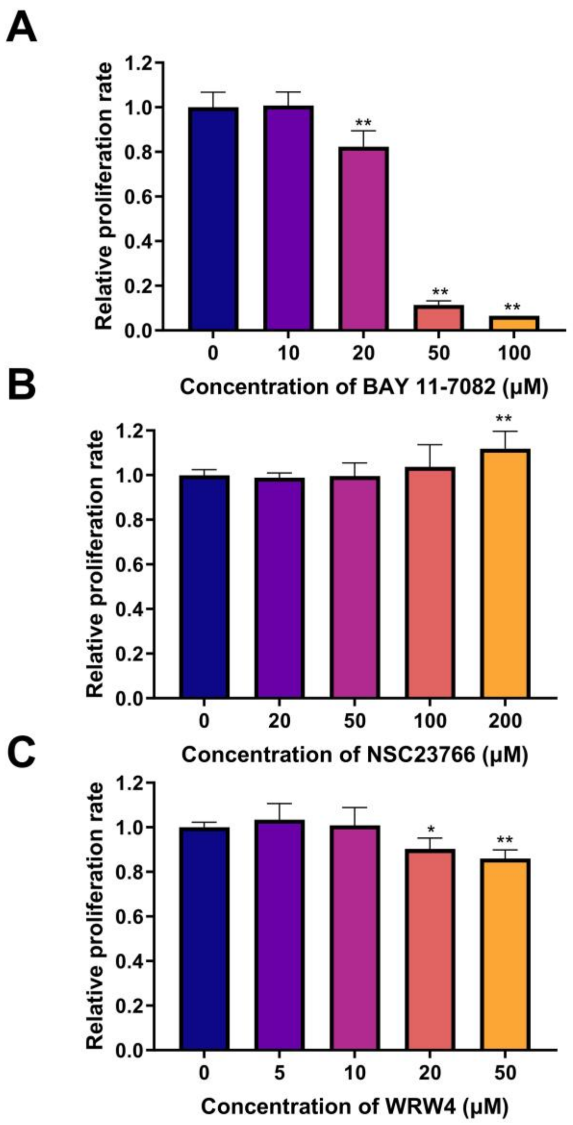

Figure S2. The effect of inhibitors on cell proliferation. A-C Relative proliferation level of RAW264.7 macrophages treated with BAY11-7082 (NF-κB inhibitor) , NSC33766 (Rac1 inhibitor), and WRW4 (FPR2 inhibitor). * $p < 0.05$, ** $p < 0.01$ between indicated groups.

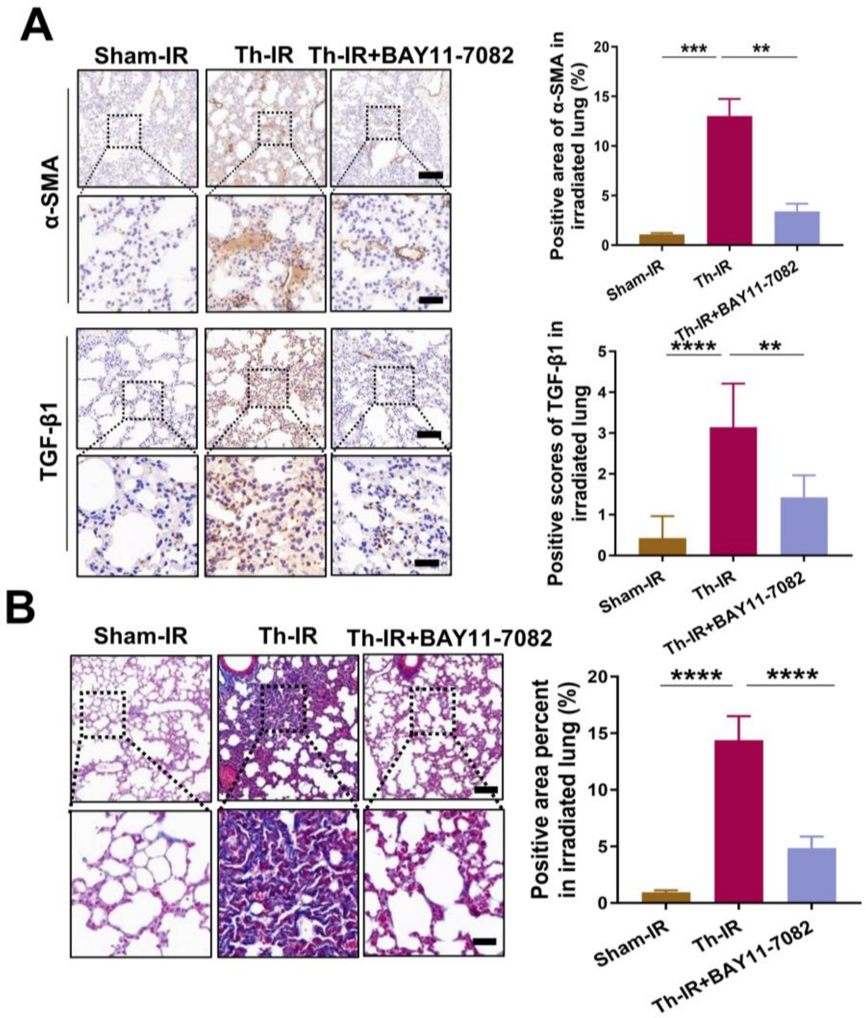

Figure S3. NF-κB mediated lung injury induced by Th-IR. **A** IHC staining for α-SMA and TGF-β1 expression in irradiated lung tissues at week 8 after Th-IR. **B** Masson trichrome staining of irradiated lung tissues at week 8. Scale bar=100 μm, magnifying scale bar=50 μm. Data were presented as mean ± SEM. ** $p < 0.01$, *** $p < 0.001$, **** $p < 0.0001$, and ns, no significant difference between indicated groups.

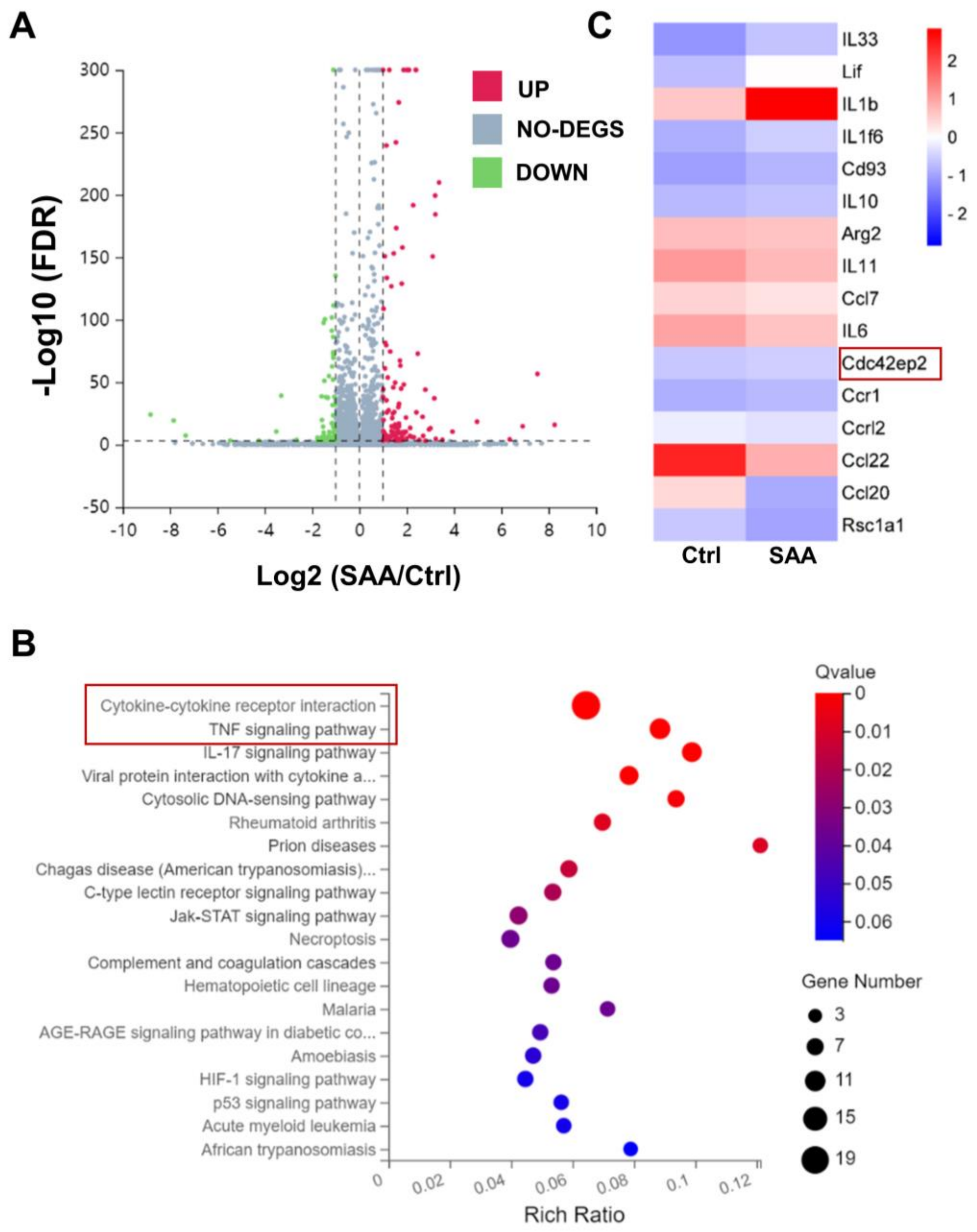

Figure S4. RNA sequencing transcriptome analysis on macrophages. **A** Volcano plots presenting the differentially expressed genes (DEGs) between 5 μg/mL SAA-treated group and control group (fold change > 2, p< 0.01). **B** Functional pathway analysis of DEGs. **C** Heatmap for differentially expressed cytokine and chemokine genes induced by SAA.

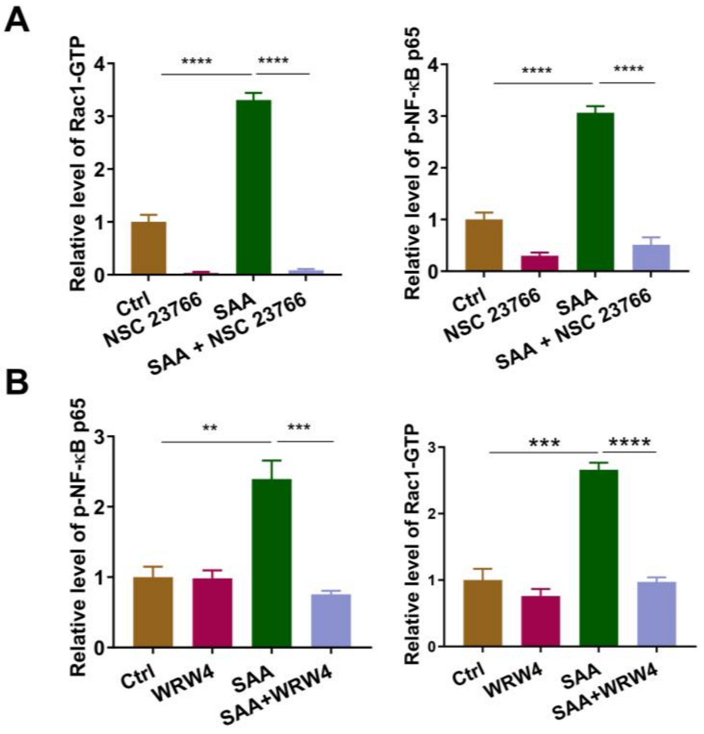

Figure S5. The statistical graphs for western blotting in Figure 4D-E. A-B The relative expression levels of p-NF-κB p65 and Rac1-GTP in macrophages after the cells being treated by NSC23766 (**A**) and WRW4 (**B**), respectively, in Figure 4D-E. ***$p < 0.01$, ****$p < 0.001$, and **** $p < 0.0001$ compared with the indicated group.

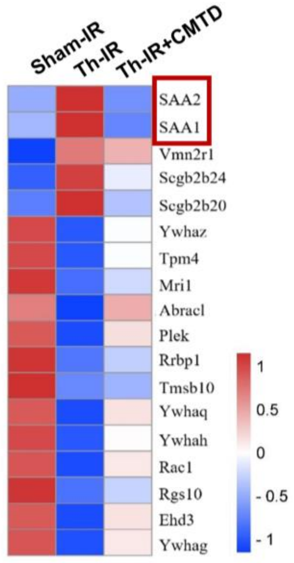

Figure S6 Heatmap of differentially expressed proteins between Sham-IR, Th-IR and Th-IR+CMTD group at day 7 after Th-IR.
